# Managing Diabetes Mellitus With Millets: A New Solution

**DOI:** 10.7759/cureus.44908

**Published:** 2023-09-08

**Authors:** Pragya Agrawal, Brij Raj Singh, Ujwal Gajbe, Minal A Kalambe, Maithili Bankar

**Affiliations:** 1 Anatomy, Datta Meghe Medical College, Datta Meghe Institute of Medical Science (Deemed to be University) Wardha, Nagpur, IND; 2 Obstetrics and Gynaecology, Datta Meghe Medical College, Datta Meghe Institute of Medical Science (Deemed to be University) Wardha, Nagpur, IND; 3 Medical Education Unit, Datta Meghe Medical College, Datta Meghe Institute of Medical Science (Deemed to be University) Wardha, Nagpur, IND

**Keywords:** millets, diabetes mellitus, glycemic index, food medicine, diet

## Abstract

Diabetes mellitus (DM) is the leading cause of morbidity and mortality, and the disease's prevalence is increasing with each passing day. DM can be prevented and controlled with modifications to the diet, especially by incorporating millet in the diet. Throughout history, eating habits have been recognized for their significant contribution to promoting health and wellness by eating foods rich in nutrients. Millet is an underutilized food crop with many benefits for health, with the most beneficial being low glycemic index, high fiber content, polyunsaturated fatty acids (PUFA), non-acid-forming potential, and gluten-free. In addition to staple food crops, such as wheat, rice, and foxtail millet, millets are still highly nutritious and beneficial and have great potential to help the world combat the food insecurity many countries face today. Millets are in the top positions of recommended dietary charts with their numerous health benefits and antioxidant properties.

## Introduction and background

In the current environment, with increasing technological access, humans have headed toward a more sedentary and stressful life, with a lack of well-balanced dietary intake and sleep. India has become a hub for diseases such as diabetes mellitus (DM), hypertension, obesity, atherosclerosis, heart diseases such as ischemic cardia, angina, and cardiac arrest, and stroke in people in much lower age groups, even in their mid-thirties and forties [1,2]. DM is characterized by a complex pathogenesis. Type 1 DM or insulin-dependent DM is an autoimmune disorder characterized by T-cell-mediated destruction of β-cells of Islets of Langerhans of the pancreas which results in a deficiency of insulin. Type 2 DM or non-insulin-dependent DM is caused either due to insulin resistance or β-cell dysfunction. Exhaustion of glucose transporters in the intestine and kidney is also a line of cause for DM [3,4]. It is a lifestyle-related disorder and can be prevented and controlled with changes in diet along with prescribed medications. Dietary interventions have played a crucial role in the treatment of DM since the golden age of the great ancient Ayurvedic practitioner Sushruta, who had testified that a person’s dietary habits, in addition to other etiological factors such as hormonal imbalance and poor dietary habits, were one of the leading causes of DM [5]. In fact, according to medical history, a diet at the level of starvation was the only treatment for this disease before the discovery of insulin [6]. Today, this carousel has changed from Allen’s starvation diet to a diet rich in carbohydrates, fats, and dietary fibers, and a high intake of millet-based dietary fiber controls glycemia, hyperinsulinemia, and lowers plasma lipids in patients with type 2 DM [5].

In addition to staple food crops, such as wheat and rice, which people have been eating for years, millet remains highly nutritious and beneficial, but it is an underutilized crop that has a multitude of benefits for health; the most beneficial are low glycemic index (GI), high fiber content, polyunsaturated fatty acids (PUFA), non-acid-forming potential, and being gluten-free [7]. Millets are nutrients rich in vitamins, minerals, proteins, essential fatty acids, energy, carbohydrates, plant chemicals, and non-glycemic polysaccharides [7-9]. Millet grains show huge benefits in their resistance to drought and high-yield production in areas with less water availability [10].

Incorporating millets into the diet along with regionally available staple food crops and vegetables has been the greatest area of interest because millets provide more significant health benefits due to their high fiber, minerals, vitamins, macro- and micronutrients, and phytochemicals and can help combat chronic disorders. Millets also have great potential to help the world combat the food insecurity that many countries are facing today [8]. Despite numerous benefits, millet consumption has been restricted only to conventional communities, poverty, and drought-stricken across the globe due to a lack of awareness among people. They are known as ‘orphan cereals’ due to their neglected use [11]. Incorporating millet into the diet may seem difficult initially, but in no time, it can become a no-brainer and a solution to numerous lifestyle-induced health problems [12]. This article aims to highlight the nutritional benefits of millet and its efficacy in controlling, managing, and preventing DM.

## Review

What are millets and their consumption over the years?

Millets are grains that are considered one of the first cultivated cereals in agricultural history. They are small-seeded cereal grasses or coarse grains that belong to the Poaceae family and are widely used as the main source of food in the arid regions of developing countries around the world and also serve as feed in developed countries [13]. It is a rain-fed crop that requires minimal irrigation and can be easily cultivated in drought-prone areas where annual precipitation is lower than normal. Millets are widely cultivated in semi-arid tropical regions of Asia, India, China, and Africa [14]. Of the total 8000 species of millets, only 35 species comprising 20 different genera have been domesticated to date for food and feed [15]. Millets are an important part of Indian cuisine and many varieties are consumed. Ragi or finger millet, bajra or pearl millet, jowar, and sorghum are among the most popular millets in India [12]. Foxtail millet, also known as kangani, is another variety that is particularly prevalent during spiritual fasts. Additionally, barnyard millet is a type of millet often consumed in Indian and Japanese cuisine. It is possible that one’s ancestors, specifically grandparents, incorporated a significant amount of millet into their diet. As evidenced by an age-old Kannada adage, ‘A rice consumer is similar to a bird in terms of weight; a jowar consumer exhibits the fortitude of a wolf; while a Ragi consumer tends to be in good health, free from diseases’ [7]. India is recognized as the leading producer of millets worldwide, and as a result, millets have been a fundamental aspect of Indian cuisine for generations. India’s annual millet production accounts for approximately 40.20% of the total [16]. The consumption of millets in the Indian diet saw a significant decline after the advent of the Green Revolution in 1965, during which rice and wheat gained wider acceptance among the populace compared to other locally grown crops [17]. In recent times, there has been renewed interest in millets within Indian agronomy, after a prolonged period of neglect.

Distribution of millets in India

India is known for its diverse milling with a series of eight different species such as foxtail millet, finger millet, small millet, barnyard millet, sorghum, kodo millet, proso millet, and pearl millet, which are growing in different regions [18]. These millets serve as a significant food crop in India, either as a main staple or as part of the seasonal rotation with other agricultural products such as pulses, spices, condiments, and oilseeds [19]. Pearl millet and sorghum are widely grown in the dry areas of Gujarat, Rajasthan, Haryana, and Uttar Pradesh [20]. Sorghum is a major crop in parts of central India, including Telangana, Maharashtra, and Andhra Pradesh, where it is grown as one of the main food crops [21]. In Tamil Nadu and Gujarat, finger millet is very often cultivated. Despite India's rich cereal diversity, the consumption of cereals has declined over the past decades.

Nutritional benefits of millets

Bioactive Compounds Present in Millets

**Figure 1:**
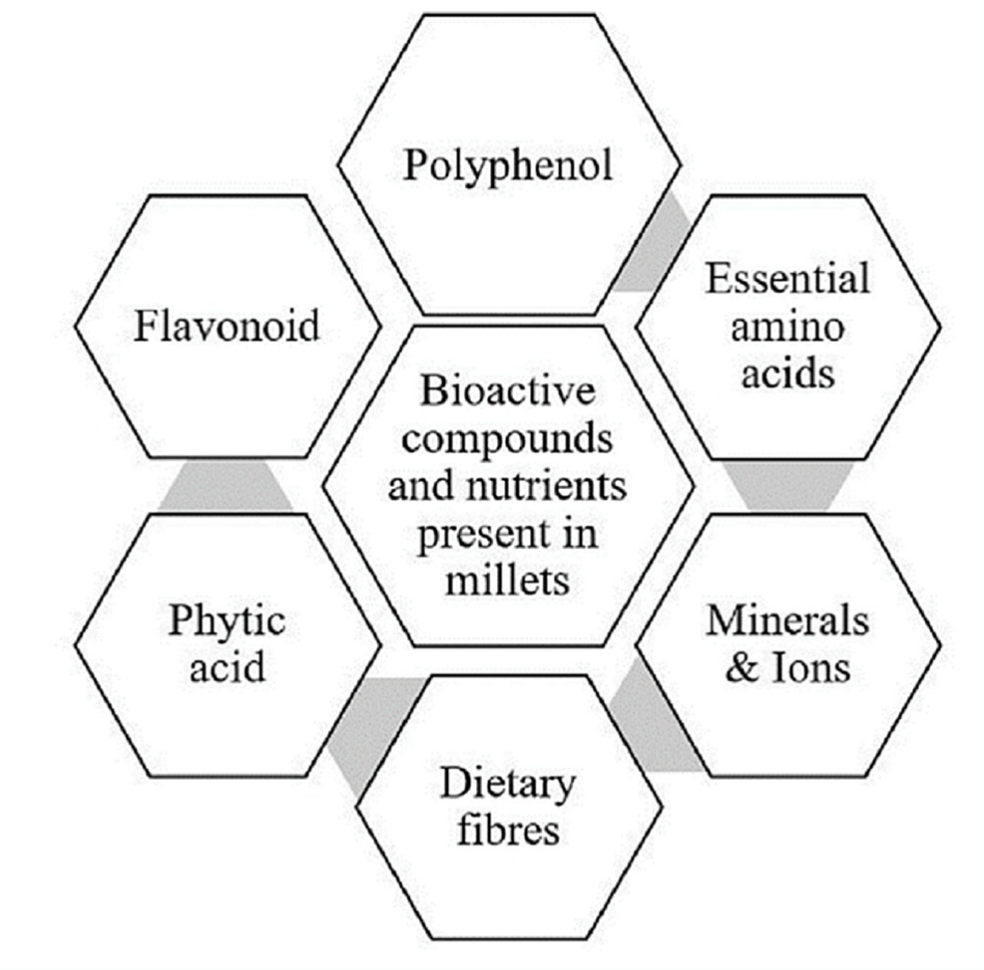
Bioactive Compounds and Nutrients Present in Millet

Millet’s seed coat, commonly known as bran, contains a significant concentration of essential nutrients, dietary fibers, and bioactive compounds such as tannins [22]. A diet regimen that incorporates a significant amount of finger millet seed coat has been shown to confer various health benefits. These include reducing inflammation, maintaining a healthy plasma lipid profile, alleviating oxidative stress, modulating the expression level of several obesity-related genes, and increasing the beneficial bacteria population of the gastrointestinal tracts such as lactic acids and bifidobacterial in a mice-based study [23]. The seed cover contains minerals such as calcium, magnesium, iron, zinc, and phosphorus, as well as globulin, albumin, and prolamin. Millets are silos of rich bioactive compounds that support the body, as shown in Figure 1.

The polyphenols present in the millet seed layer enrich them with antioxidant properties along with anti-aspirin properties. A large amount of phytic acid present in millets, especially finger millets (ragi), reduces carbohydrate digestibility and mitigates postprandial blood glucose levels [24]. Therefore, finger millet has a potential as a food option for diabetics. Finger millet contains twice the amount of calcium in milk and 10 times more calcium than brown rice, wheat, and corn. Consumption of finger millet during and after pregnancy and lactation can provide significant benefits to maternal and child bone health and prevent osteoporosis. Calcium provides structure and rigidity to the body and mediates vascular and muscular contractions and nerve signal transmission [25-27]. Therefore, a balanced calcium dietary intake is recommended because excessive calcium is linked with mortality and causes vascular events in people receiving calcium supplementation [28,29]. The prebiotic components inherent in millets are metabolized by indigenous bacteria in the human gut to produce beneficial short-chain fatty acids and probiotics from the colon, which have been shown to possess antidiabetic properties [30]. Foxtail millet is particularly rich in resistant starch, which has the ability to delay gastric emptying and decrease post-consumption blood glucose levels [31]. Sorghum is enriched with prolamin (kefirin), a protein that, when cooked, becomes comparatively less digestible compared to proteins present in other cereals [32]. Pearl millet (bajra) is rich in zinc, iron, dietary fibers, and omega-3 fatty acids, which provide antioxidant properties when consumed [14]. Fibers rich in millets show a positive change in glycol-lipid parameters. So, they turned out to be the best rice alternative. Millets possess a significant concentration of critical amino acids, including phenylalanine, methionine, leucine, and isoleucine. In addition to these, they contain enough concentrations of vitamin E, vitamin B, calcium, iron, proteins, minerals, riboflavin, and thiamine present in them. Kodo millets act as a barrier to hyperlipidemia due to their high lecithin content and better seed viability [33]. Gluten-free teff millet can be used as an alternative to bakery items. Proso millet is a reservoir of lecithin, B-complex vitamins, and critical amino acids that promote neural health [34].

Benefits of Millets for Various Health Ailments 

Millets provide an approximate energy yield of 320-370 kcal per 100 g of millet consumption [35]. These substances exhibit the properties of antioxidants, immune modulators, and detoxifiers. They improve gastrointestinal and cardiovascular health and prevent the occurrence of cancers, celiac diseases, diabetes, hypertension, hyperlipidemia, duodenal ulcers, Parkinson’s disease, etc. [8,36]. Researchers have reported that millet intake increases the amount of high-density lipoproteins in plasma and regulates cholesterol metabolism [12,37]. Regular millet consumption can reduce the incidence of hormone-dependent cancers, such as breast cancer, and reduce the risk of cardiovascular disease in post-menopausal women. Millets are also known to slow the process of aging in humans [37]. Millets have higher levels of essential amino acids. Pearl millet is rich in arginine, threonine, valine, isoleucine, and leucine. Finger millet contains a balanced amount of essential amino acids. It contains higher amounts of lysine, valine, and threonine as compared to other varieties of millet. Proso millet contains the essential amino acids in significantly larger quantities, except for lysine. Little millet is a rich source of sulfur-containing amino acids including cysteine and methionine. The presence of these amino acids in millets indicates the potential benefits of millets in human health [38]. The health benefits of the most popular millets found in the Indian subcontinent are listed in Table 1.

**Table 1:** Health Benefits of Different Types of Millets

Variety of millet	Health benefits
Foxtail millet (kangani)	Helps combat diseases such as osteoporosis and fractures because they are rich in calcium and proteins [39].
Sorghum (jowar)	Contains antioxidants that reduce the risk of cardiovascular disease and colon cancer. Their highly soluble fibers reduce the risk of type II diabetes [33].
Barnyard millet (sanwa rice)	Accelerates hemoglobin production and maintains healthy red blood cells because they are rich in iron [12].
Pearl millet (bajra)	Protects body tissues from free radical damage because they are rich in vitamin E. Suitable for people with gluten sensitivity [14].
Finger millet (ragi)	Helps to strengthen and develop bones and prevent anemia because they are rich in calcium and polyphenols [26].
Little millet (sama)	Prevents spikes in blood glucose levels and helps control diabetes. It also prevents heart disease [8].
Kodo millet	Excellent aid in the health of the nervous system. It is very easily digested and is used for the formulation of infant and geriatric products [33].
Proso millet	Controls depression, lowers blood pressure, and acts as an anti-inflammatory agent. They are the millet of choice for cardiac patients because they are rich in thiamine and energy [33].

The GI of millet

The GI is the ability of foods to change blood glucose levels. Foods with higher GIs cause rapid increases in blood sugar levels, whereas foods with lower GIs cause gradual or steady increases in blood sugar levels. Seventeen studies out of a systematic review of 19 studies have shown a general reduction not only in blood sugar levels but also in serum cholesterol and serum triglycerides in patients who consume foods lower on the side of the GI [12]. Millets show a score between 40 and 70 on the GI chart, which is less than the GI value of wheat, refined flour, rice, and maize [40]. The consumption of proso millets showed a lower GI than those produced with wheat and corn. The GI and protein composition of different varieties of millet shown in India are shown in Table 2 [41]. 

**Table 2:** Glycemic Index and Protein Composition of Different Millet Varieties

Millet variety	Glycemic index	Protein content
Foxtail millet	50-60	12.3%
Little millet	50-65	7.7%
Kodo millet	50-65	8.3%
Proso millet	50-65	11.5%
Barnyard millet	50-65	11.2%
Finger millet	70-80	7.3%
Pearl millet	70-85	10.6%
Sorghum	70-85	10.4%
Amaranth	65-70	13.6%

Food medicine for DM

DM is characterized by a disturbance in body glucose homeostasis along with a disturbance in the levels of carbohydrates, proteins, and fats in the body. Hyperglycemia not only results in DM but the onset of DM paves the way for the development of many diseases along with it. A common feature of many non-communicable diseases is mitochondrial dysfunction as an underlying pathology. Mitochondrial perturbations are upstream of insulin resistance [42]. The most common ones include atherosclerosis, diabetes retinopathy, fractures due to demineralization, muscle fatigue, and renal problems [43]. Research has shown that diabetics have higher chances of kicking the bucket due to respiratory failure and multiple organ failure than non-diabetics [44]. Today, when India has become the most populous nation in the world, at the same time nearly 8-10% of its diabetic population is in the age group of 20-70 years, which is becoming a liability for a country like ours where a maximum of its population falls under the category of youth. India is considered the diabetic capital of the world [45]. It has been predicted that within the next decade, the number of cases of diabetes will exceed 350-450 million [37]. There has been a significant increase in cases of diabetes throughout the world due to an immense increase in population, aging, urbanization, increased obesity, and decreased physical activity.

Therefore, diet management is the central and most economical way to manage DM and the complications associated with it. Various diabetes associations across the globe offer guidelines for the consumption of adequate amounts of carbohydrates, proteins, fats, fibers, and sodium in the diet to promote healthy eating habits among individuals with diabetes. According to the American Diabetes Association (ADA), it is recommended that carbohydrates contribute 45-60% of the total calories, proteins 15-20%, and fats 25-35%. Also, a minimum of 14 g of fiber per 1,000 calories should be consumed, while at the same time, sodium intake should be limited to less than 2,300 mg per day. Similarly, the Canadian Diabetes Association (CDA) suggests a comparable distribution of macronutrients, with an emphasis on a daily fiber intake of 25-50 g. The British diabetes guidelines are close to the ADA recommendations, although they propose a slightly lower fat intake of 30% or less of total calories. The European Diabetes Research Association (EASD) also promotes similar guidelines for carbohydrate, protein, and fat intake, highlighting a daily fiber consumption of at least 25 g and restricting sodium intake to 2,000 to 2,400 mg per day. These associations strive to provide comprehensive nutritional guidance to people with diabetes, advocating for a balanced diet that supports general health and aids in blood sugar control, as shown in Table 3 [46].

**Table 3:** Recommended Dietary Intake by Leading Health Associations g, grams; mg, milligrams.

Association	Carbohydrate intake	Protein intake	Fat intake	Fiber intake	Sodium intake
American Diabetes Association (ADA)	45-60% of total calories	15-20% of total calories	25-35% of total calories	At least 14 g per 1,000 calories	Less than 2,300 mg per day
Canadian Diabetes Association (CDA)	45-60% of total calories	15-20% of total calories	20-35% of total calories	At least 25-50 g per day	Less than 2,300 mg per day
Diabetes United Kingdom (UK)	45-60% of total calories	10-20% of total calories	30% or less of total calories	At least 30 g per day	Less than 2,400 mg per day
European Association for the Study of Diabetes (EASD)	45-60% of total calories	15-20% of total calories	25-35% of total calories	At least 25 g per day	Less than 2,000-2,400 mg per day

Millets stand by as the ideal food crop for people with diabetes according to the criteria set by leading associations. The high fiber content and phenolic content of the diet make millet, especially foxtail millet, very fruitful for DM [37]. Diabetics show a major sign of polyphagia and frequent food cravings. Millets reduce the duration of gastric emptying to maintain constant postprandial body glucose homeostasis [47]. Polyphenolic ligands have an inhibitory effect on alpha-glucosidase and pancreatic amylases to reduce postprandial hyperglycemia by inhibiting the enzyme hydrolysis of complex carbohydrates [48,49]. Millets contain slowly digested starch that extends the digestion and absorption of carbohydrates in the intestine. Compared to widely consumed rice, millet releases less glucose into the blood for a longer period of time, which is attributable to diabetes prevention. Millets help in the management of body weight, which is of utmost importance in diabetic patients. Pearl millet increases insulin sensitivity and reduces triglyceride levels in the body.

How to incorporate millet into the diet

Millets are the cereals of today that can be very easily mixed into a diet. Initially, a low amount of millet should be incorporated into the diet with a gradual increase over the period. Traditionally, millets were puffed, flaked, and popped to be consumed in the diet [50]. Germinating and fermenting millet improve its nutrient availability while, at the same time, excessive polishing, grinding, and dehulling reduce its nutrient quality. Millets can be consumed at any time of the day. People are of the misconception that healthy food cannot be tasty. Millets break these misconception shackles and can be consumed with any food item of choice. Millets can be replaced with rice in the diet and compensate for any delicacy that requires rice as its main ingredient. A portion of millet can not only be consumed as salad or soup, but millet flour can be used to make chapattis (flattened Indian bread), dosas (fermented Indian bread), and bread to eat with vegetables, legumes, and pulses of choice. Porridge and kheer (traditional Indian preparation made with milk) made from millets, especially pearl millets, are widely consumed in the Rajasthan and Gujarat regions during winter [51]. Recent times have seen the emergence of noodles, vermicelli, pasta, bakery products, and sweets made of millet, especially finger millet [27]. One should try various varieties of millets available on the market, then decide which one suits the body the most.

## Conclusions

Millets are ‘future crops’ that have the potential to merge as a powerful and effective solution to various metabolic diseases such as DM due to the high levels of micronutrients and macronutrients present in them. There remains an urgent need to develop more technologies to overcome the antinutrient property of millets to improve the industrial-scale processing of millets. Physicians, nutritionists, and patients themselves should try to incorporate millet into their diet and follow a strict, balanced, and planned diet in combination with regular exercise or walking. No single food provides 100% nutrients, so it is a prerequisite to incorporate them wisely into our diet in combination with other food sources. A meal comprising a combination of pulses, millet, and functional foods is the ideal meal.
